# Independent Evaluation of the Integrated Community Case Management of Childhood Illness Strategy in Malawi Using a National Evaluation Platform Design

**DOI:** 10.4269/ajtmh.16-0110b

**Published:** 2016-06-01

**Authors:** Agbessi Amouzou, Mercy Kanyuka, Elizabeth Hazel, Rebecca Heidkamp, Andrew Marsh, Tiope Mleme, Spy Munthali, Lois Park, Benjamin Banda, Lawrence H. Moulton, Robert E. Black, Kenneth Hill, Jamie Perin, Cesar G. Victora, Jennifer Bryce

**Affiliations:** UNICEF, New York, NY. E-mail: aamouzou@unicef.org; National Statistical Office, Zomba, Malawi. E-mail: mkanyuka@gmail.com; Institute for International Programs, Johns Hopkins Bloomberg School of Public Health, Baltimore, MD. E-mails: ehazel1@jhu.edu, rheidka1@jhu.edu, and amarsh6@jhmi.edu; National Statistical Office, Zomba, Malawi. E-mail: tmleme@yahoo.cu.uk; Chancellor College, University of Malawi, Zomba, Malawi. E-mail: spymunthali@gmail.com; Institute for International Programs, Johns Hopkins Bloomberg School of Public Health, Baltimore, MD. E-mail: lpark9@jhu.edu; National Statistical Office, Zomba, Malawi. E-mail: bisa1banda@yahoo.com; Institute for International Programs, Johns Hopkins Bloomberg School of Public Health, Baltimore, MD. E-mails: lmoulto1@jhu.edu, rblack1@jhu.edu, kenneth_hill_1@yahoo.com, and jperin@jhu.edu; University of Pelotas, Pelotas, Brazil. E-mail: cvictora@gmail.com; Institute for International Programs, Johns Hopkins Bloomberg School of Public Health, Baltimore, MD. E-mail: jbryce1@jhu.edu

Dear Sir:

Doherty and others suggest that our evaluation of the impact of the integrated Community Case Management of childhood illness (iCCM) in Malawi did not take into account spatial variations in exposure to iCCM within districts, thereby diluting the measured impact.^1^ We find this critique unfounded given the purpose of our evaluation.

The iCCM strategy as implemented by the Ministry of Health and partners in Malawi aimed to improve intervention coverage and reduce under-five mortality among all children. Central components of the strategy were to complement existing facility-based health services with trained community health workers in district-defined “hard-to-reach areas” (HTRAs), while simultaneously strengthening the quality of facility-based child health care, referral, commodities distribution, and supervision. An evaluation focused only on the effects of deploying community-based workers would miss the potential contribution of these broader health system interventions, as well as any negative side effects of the iCCM strategy such as the displacement of program effort from easily accessible areas to HTRAs. The appropriate unit of analysis for the evaluation is therefore the district, with all under-five children included in the analysis.

In addition, Doherty and others' recommendations for alternative evaluation designs are flawed, and suggest an unfamiliarity with the realities of large-scale evaluations. Their suggestion to stratify the analysis by a measure of spatial exposure such as distance from a household to a health surveillance assistant (HSA) or to estimate a ratio of HSAs per population in a specified area ignores the fact that neither these data nor geospatial boundaries of district-defined HTRAs is available in Malawi. Our evaluation did examine the effects of iCCM among rural populations and the poorest 20%, and found no substantial change in care seeking over time (see Figure 3 and Supplemental Webannex 1, Part 8 in the article).

The authors of the letter also suggest that using change in care seeking from HSAs as the dependent variable would produce “more accurate results” relative to the measure of overall care seeking used in our evaluation. We not only report these results (an increase in care seeking from iCCM-HSAs from 2% to 10% during the implementation period), but also document that this increase was largely as a result of mothers changing their source of care from a fixed health facility to an iCCM HSA. Limiting the analysis to those seeking care from HSAs would miss this replacement effect and also produce unstable results due to small sample sizes.

We agree that it would be preferable to use correct treatment rather than care seeking as an intermediate measure. Unfortunately, there is now convincing evidence that household surveys cannot produce accurate measures of treatment of childhood pneumonia, and care seeking provides the best available measure until new methods are developed and tested.^2^–^4^

In sum, an evaluation approach that defines exposure at HTRAs level would not measure the impact of roll-out of the iCCM at population level in districts or nationally. Such approach would answer a different and much more limited question—that is, what are the effects of iCCM in HTRAs as compared with non-HTRAs? Although answering this question might demonstrate the effectiveness of iCCM as a stand-alone service delivery approach, it does not provide a test of the full program strategy and Malawi's intent to reduce child mortality at district and national levels.

Our rigorous approach has provided important evidence to the government of Malawi that they are using to revise and strengthen implementation of iCCM strategy in the context of their overall child survival strategy. Doherty and others' concern that the evaluation results may lead to reductions in funding for iCCM and demotivation of program managers is unjustified and inappropriate, implying that the reporting of evaluation results should be tempered to reinforce the status quo.

## References

[1] Amouzou A, Kanyuka M, Hazel E, Heidkamp R, Marsh A, Tiope M, Munthali S, Lois P, Banda B, Moulton LH, Black RE, Hill K, Victora CG, Bryce J (2016). Independent evaluation of the integrated Community Case Management of childhood illness strategy in Malawi using a National Evaluation Platform design. Am J Trop Med Hyg.

[2] Campbell H, el Arifeen S, Hazir T, O'Kelly J, Bryce J, Rudan I, Qazi SA (2013). Measuring coverage in MNCH: challenges in monitoring the proportion of young children with pneumonia who receive antibiotic treatment. PLoS Med.

[3] Hazir T, Begum K, el Arifeen S, Khan AM, Huque MH, Kazmi N, Roy S, Abbasi S, Rahman QS, Theodoratou E, Khorshed MS, Rahman KM, Bari S, Kaiser MMI, Saha SK, Ahmed AS, Rudan I, Bryce J, Qazi SA, Campbell H (2013). Measuring coverage in MNCH: a prospective validation study in Pakistan and Bangladesh on measuring correct treatment of childhood pneumonia. PLoS Med.

[4] Geldsetzer P, Williams TC, Kirolos A, Mitchell S, Ratcliffe LA, Kohli-Lynch MK, Bischoff EJL, Cameron S, Campbell H (2014). The recognition of and care seeking behaviour for childhood illness in developing countries: a systematic review. PLoS One.

